# Coronary occlusion after the Manouguian procedure in a patient with a single coronary artery: a case report

**DOI:** 10.1186/s13019-016-0542-8

**Published:** 2016-10-24

**Authors:** Yochun Jung, Byoung Hee Ahn, Kyo Seon Lee, In Seok Jeong, Sang Gi Oh, Kook Joo Na, Kye Hun Kim

**Affiliations:** 1 Department of Thoracic and Cardiovascular Surgery, Chonnam National University Hospital, Chonnam National University School of Medicine, 42 Jebong-ro, Dong-gu, Gwangju 501-757 South Korea; 2 Department of Thoracic and Cardiovascular Surgery, Chonnam National University Hwasun Hospital, Chonnam National University School of Medicine, Hwasun, South Korea; 3 Department of Cardiovascular Medicine, Chonnam National University Hospital, Chonnam National University School of Medicine, Gwangju, South Korea

**Keywords:** Aortic valve replacement, Single coronary artery, Coronary artery occlusion, Case report

## Abstract

**Background:**

The association between the anatomy of a single coronary artery (SCA) and the surgical risk of aortic valve replacement (AVR) remains unclear due to a lack of studies on this topic.

**Case presentation:**

A 73-year-old woman underwent AVR for aortic stenosis. Preoperative coronary angiography results showed a SCA arising from the left coronary sinus. The Manouguian procedure was performed for a small aortic annulus. Intraoperatively, an extracorporeal membrane oxygenator (ECMO) was needed for bypass weaning failure due to newly developed right ventricular dysfunction. Coronary angiography was performed on postoperative day 4, and the findings showed a right coronary artery occlusion just after its origin. After emergent coronary artery bypass surgery, she could be weaned from the ECMO. She was discharged on postoperative day 70 and followed up without complications for 12 months.

**Conclusions:**

AVR with the annular enlargement procedure in those with a SCA can result in an unexpected coronary artery occlusion, which should be, therefore, suspected when unexplained myocardial dysfunction occur. For reducing this risk, the use of a small prosthesis should be considered over the annular enlargement procedure when performing AVR in those with a small aortic annulus and a SCA.

**Electronic supplementary material:**

The online version of this article (doi:10.1186/s13019-016-0542-8) contains supplementary material, which is available to authorized users.

## Background

The prevalence rate of isolated single coronary artery (SCA) ranges from 0.024 to 0.066 % [1, 2]. The association between the anatomy of a SCA and the surgical risk of aortic valve replacement (AVR) remains unclear due to a lack of studies on this topic. The present report describes the occurrence of a coronary artery occlusion after AVR was performed with the annular enlargement procedure for a small aortic annulus in a patient with a SCA.

## Case presentation

**Fig. 1:**
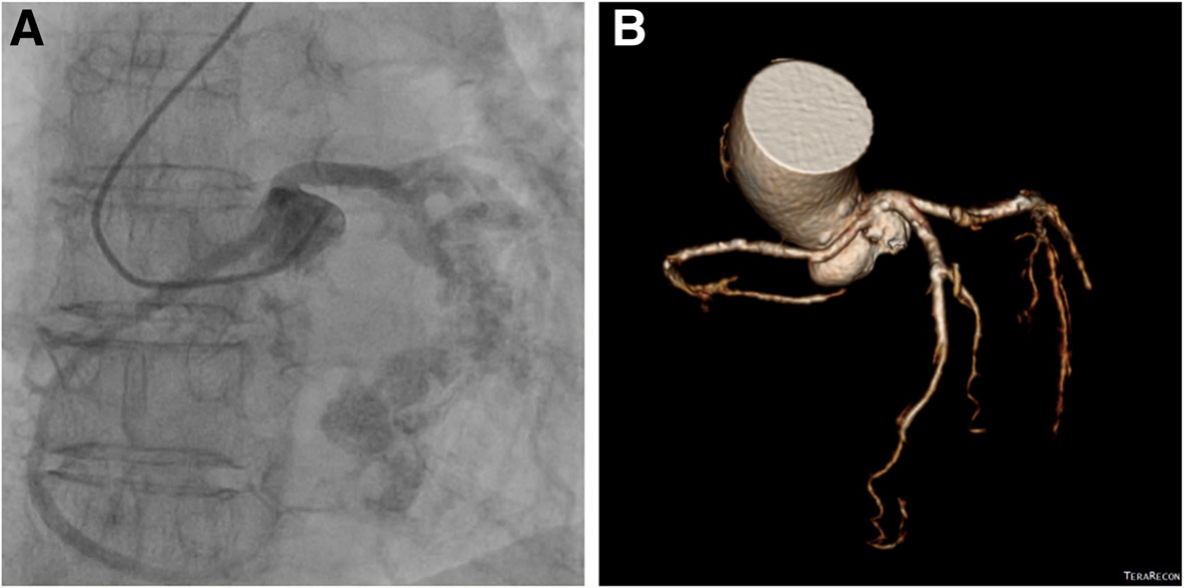
Preoperative imaging. **a** Coronary angiography images. **b** Computed tomography coronary angiography images. A type LII-B single coronary artery was observed according to Lipton’s classification

A 73-year-old woman underwent AVR for aortic stenosis. Preoperative coronary angiography (CAG) results showed a SCA originating from the left coronary sinus (Lipton’s classification LII-B) without any stenotic lesion (Fig. 1a, b). The operation was conducted under moderate hypothermia. After cross-clamping the ascending aorta, cardiac arrest was induced by infusing a cold blood cardioplegic solution through the coronary sinus. The cardioplegic solution was infused in the same manner every 30 min during the surgery. The aortic valve was tricuspid and degenerative; a single coronary ostium was observed in the left coronary sinus. The aortic annulus was tighter than expected when it was sized, thus it was difficult to implant the 21-mm Hancock II valve (Medtronic, Minneapolis, MN). Therefore, during implantation of the prosthetic valve, unplanned Manouguian procedure was performed to enlarge the annulus. Subsequently, AVR was finished without further difficulties. We ensured that the coronary ostium was not blocked by a valve strut prior to closing the aortotomy. Thirty minutes after weaning cardiopulmonary bypass (CPB), gross distension and reduced contractility of the right atrium and right ventricle were noticed, so CPB was resumed. Intraoperative transesophageal echocardiography results showed the same finding and a newly developed tricuspid regurgitation; the left ventricular contractility was normal, and the prosthetic aortic valve functioned well. Presuming that the right ventricular dysfunction was due to issues with myocardial protection in the right coronary artery (RCA), we tried to wean CPB more slowly. After 2 more hours of unsuccessful CPB weaning, we implemented an extracorporeal membrane oxygenator (ECMO) via femoral cannulae to allow more time for myocardial recovery, and then CPB weaning was performed to complete the operation. The aortic cross-clamp time and total CPB time were 144 and 324 min, respectively.

**Fig. 2:**
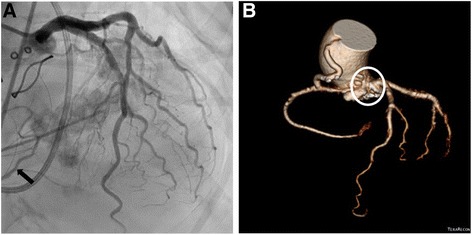
Postoperative imaging. **a** Coronary angiography images on postoperative day 4. The origin of the right coronary artery was not observed; however, the distal right coronary artery was seen because of backflow (*arrow*). **b** Computed tomography coronary angiography images. The coronary artery bypass graft was observed from the ascending aorta to the proximal right coronary artery, and the proximal right coronary artery was not visible (*white circle*)

Postoperatively, the serum creatine kinase-MB level increased to 82.2 ng/dL on postoperative day (POD) 1, but it decreased rapidly to normal levels by POD 3. Additionally, there were no signs of potential myocardial ischemia observed on electrocardiography examination. However, ECMO weaning was unsuccessful, and follow-up echocardiography findings showed newly developed ventricular dysfunction on the left side in addition to persistent right ventricular dysfunction. On POD 4, CAG was performed to make a differential diagnosis, and the findings showed total occlusion of the origin of the RCA branching off from the common coronary trunk, resulting in backflow through the collateral vessels; blood flow of the left coronary artery was normal (Fig. 2a). Percutaneous coronary intervention was attempted to recanalize the RCA, but the guide wire could not be passed due to the unusual angulation of the course of the RCA. Hence, we performed emergency bypass grafting from the ascending aorta to the proximal RCA by using a saphenous vein graft (Fig. 2b).

Postoperative follow-up echocardiography results indicated functional improvement in both ventricles, and the patient was weaned from the ECMO on POD 10. She was discharged on POD 70, and she is undergoing outpatient follow-up without complications for 12 months.

## Discussion

This is the first report of a case in which performing AVR with the Manouguian procedure caused a coronary occlusion in a patient with a SCA. Previous studies have reported successful AVR [3] or aortic root replacement [4] in patients with a SCA, and more recently, successful transcatheter aortic valve implantation was reported [5]. However, there have been no previous case reports on the annular enlargement procedure in a patient with a SCA; therefore, the mechanism that caused a coronary occlusion after this procedure is unknown. Our patient’s common coronary trunk originated from the left coronary sinus; the RCA was derived from the most proximal part of the common coronary trunk, and it passed anteriorly to the right coronary sinus and toward the right side (Lipton’s classification LII-B). We think that during the Manouguian procedure, a clockwise external force was directly applied to the left coronary sinus, which led to a delicate distortion of the common coronary trunk and caused the resultant occlusion of the proximal RCA. Therefore, in all types of SCA where the common coronary trunk originates from the left coronary sinus (i.e., types LI, LII-A, LII-B, and LII-P according to Lipton’s classification), the Manouguian procedure will likely expose patients to the risk we describe herein. Theoretically, other annular enlargement procedures such as Nicks and Konno procedures may also affect the geometry of the aortic root, which should be investigated further. Thus, based on our experience, we think that when performing AVR in patients with a SCA and a small aortic annulus, the use of a small prosthesis should be considered over the annular enlargement procedure. In cases where annular enlargement is unavoidable, the best efforts, even aortic root replacement, should be taken to minimize external force on the coronary ostium according to the type of SCA to avoid coronary occlusion.

Looking back, it took us a long time to determine that the coronary occlusion caused the intraoperative right ventricular dysfunction and postoperative ECMO weaning failure, which resulted in delayed recovery for the patient. There were several reasons for this delayed diagnosis. Intraoperative assurance of coronary ostial patency made us overly confident that no coronary occlusion would occur, thus we disregarded this coronary complication as a cause from the beginning. Moreover, neither electrocardiography results nor postoperative cardiac enzyme changes showed any suggestive findings of myocardial ischemia, and no definite regional wall motion abnormality of the left ventricle was identified by intraoperative transesophageal echocardiography. Therefore, one of the key messages of this study is that clinicians should consider coronary occlusion when unexpected myocardial dysfunction occurs after the annular enlargement procedure has been conducted in a patient with a SCA, because the usual signs of myocardial ischemia cannot be eminently detected under ECMO support even if there is total occlusion of the coronary artery.

## Conclusions

AVR with the annular enlargement procedure in those with a SCA can result in an unexpected coronary artery occlusion, which should be, therefore, suspected when unexplained myocardial dysfunction and vital instability occur. For reducing this risk, the use of a small prosthesis should be considered over the annular enlargement procedure when performing AVR in those with a small aortic annulus and a SCA.

## Additional file

Additional file 1: CARE Checklist – 2016: Information for writing a case report (DOCX 490 kb)

## Abbreviations

AVR: Aortic valve replacement
CAG: Coronary angiography
CPB: Cardiopulmonary bypass
ECMO: Extracorporeal membrane oxygenator
POD: Postoperative day
RCA: Right coronary artery
SCA: Single coronary artery
