# Supplementary material for: Coronary occlusion after the Manouguian procedure in a patient with a single coronary artery: a case report
Source: J Cardiothorac Surg. 2016 Oct 24;11:150. doi: 10.1186/s13019-016-0542-8 (PMC5078910; doi:10.1186/s13019-016-0542-8)
Supplement: Additional file 1: — CARE Checklist – 2016: Information for writing a case report (DOCX 490 kb) [file 13019_2016_542_MOESM1_ESM.docx]

**CARE Checklist – 2016: Information for writing a case report**

**Topic Item Checklist item description Line/Page**

**Title 1** The words “case report” should be in the title along with the area of focus 2 / 1

**Key Words 2** Four to seven key words—include “case report” as one of the key words 18, 19 / 2

**Abstract 3a** Background: What does this case report add to the medical literature? 2,3 / 2

**3b** Case summary: chief complaint, diagnoses, interventions, and outcomes 4-11 / 2

**3c** Conclusion: What is the main “take-away” lesson from this case? 12-16 / 2

**Introduction 4** The current standard of care and contributions of this case—with references (1-2 paragraphs) 2-6 / 3

**Timeline 5** Information from this case report organized into a timeline (table or figure) _N/A___

**Patient Information 6a** De-identified demographic and other patient or client specific information _N/A___

**6b** Chief complaint—what prompted this visit? _N/A___

**6c** Relevant history including past interventions and outcomes _N/A___

**Physical Exam 7** Relevant physical examination findings _N/A___

**Diagnostic 8a** Evaluations such as surveys, laboratory testing, imaging, etc. 9-11 / 3

**Assessment 8b** Diagnostic reasoning including other diagnoses considered and challenges 19 / 3 - 6 / 4

**8c** Consider tables or figures linking assessment, diagnoses and interventions 9-15 / 4

**8d** Prognostic characteristics where applicable _N/A___

**Interventions 9a** Types such as life-style recommendations, treatments, medications, surgery 12-15 / 4

**9b** Intervention administration such as dosage, frequency and duration _N/A___

**9c** Note changes in intervention with explanation _N/A___

**9d** Other concurrent interventions _N/A___

**Follow-up and 10a** Clinician assessment (and patient or client assessed outcomes when appropriate) 18 / 4

**Outcomes 10b** Important follow-up diagnostic evaluations 16-17 / 4

**10c** Assessment of intervention adherence and tolerability, including adverse events _N/A___

**Discussion 11a** Strengths and limitations in your approach to this case _N/A___

**11b** Specify how this case report informs practice or Clinical Practice Guidelines (CPG) 10-26 / 5

**11c** How does this case report suggest a testable hypothesis? 25 / 4 –10 / 5

**11d** Conclusions and rationale 3-7 /6

**Patient Perspective 12** When appropriate include the assessment of the patient or client on this episode of care _N/A___

**Informed Consent 13** Informed consent from the person who is the subject of this case report is required by most journals 7,8 /8

**Additional Information 14** Acknowledgement section; Competing Interests; IRB approval when required 2 /8 – 7 / 9
